# Milling Microchannels in Monel 400 Alloy by Wire EDM: An Experimental Analysis

**DOI:** 10.3390/mi11050469

**Published:** 2020-04-29

**Authors:** Mustafa Saleh, Saqib Anwar, Abdualziz El-Tamimi, Muneer Khan Mohammed, Shafiq Ahmad

**Affiliations:** 1 Industrial Engineering Department, College of Engineering, King Saud University, Riyadh 11421, Saudi Arabia; sanwar@ksu.edu.sa (S.A.); atamimi@ksu.edu.sa (A.E.-T.); ashafiq@ksu.edu.sa (S.A.); 2 Raytheon Chair for Systems Engineering (RCSE Chair), Advanced Manufacturing Institute, King Saud University, Riyadh 11421, Saudi Arabia; muneerkm@ksu.edu.sa

**Keywords:** wire electrical discharge machining (EDM), micromachining, microchannel, Monel 400

## Abstract

This paper presents the results of an investigation on the capacity of wire electrical discharge machining (WEDM) to produce microchannels in the Nickel-based alloy, Monel 400. The main objective of the current study is to produce microchannels with desired/target geometry and acceptable surface quality. Square cross-sectional microchannels with dimensions of 500 × 500 µm were investigated. Experiments were conducted based on the one-factor-at-a-time approach for the key input WEDM process parameters, namely pulse-on time (TON), pulse-off time (TOFF), average gap voltage (VGAP), wire feed (WF), and dielectric flow rate (FR). Dimensional accuracy, machining speed, surface roughness, surface morphology, microhardness, and microstructure were analyzed to evaluate the microchannels. The minimum errors of 6% and 3% were observed in the width and depth of the microchannels, respectively. Furthermore, microchannels with enhanced surface integrity could be produced exhibiting smooth surface morphology and shallow recast layer (~0–2.55 µm).

## 1. Introduction

Nowadays, nickel-based alloys, such as Monel 400 alloy, find widespread applications, including nuclear processing reactors, heat exchangers and other major industries. Furthermore, it is extensively used in marine engineering, chemical, and hydrocarbon processing equipment, pumps, valves, and sensors [1]. This is due to its high strength and toughness, the ability to resist cavitation erosion over wide temperature ranges and excellent resistance to many corrosive environments [2]. Generally, in nickel-base alloys, the machining of such alloys is hardly carried out in conventional machining processes, especially in the case of machining microfeatures, due to their superior mechanical properties in addition to the lower thermal conductivity, high heat capacity and high chemical reactivity to most tool materials [3,4]. High tooling wear, poor surface quality, and poor machinability are obvious drawbacks of conventional micromilling and consequently restrict the process for precise microfeaturing [5,6]. These issues could be overcome using non-traditional machining processes such as laser beam machining (LBM), photochemical machining (PCM), abrasive water jet machining (AWJM) and wire electrical discharge machining (WEDM). Among these processes, WEDM has higher machining efficiency and could provide higher flexibility in machining regular and complex shapes and profiles with high precision for microfeatures, especially for conductive materials [7]. Additionally, it is worth mentioning that, as compared to the laser machining and photochemical machining, the WEDM process has a higher capacity to control the shape of the microchannels, because the laser produces tapered channels as demonstrated by Ahmed et al. [8] and has deep detrimental impact [9], whereas, the photochemical machining produces channels with undercuts as shown by Patil and Mudigonda [5]. In addition, the photochemical process is a slower process, not environmentally safe and can only be applied for mass production. Regarding the AWJM, it can produce microchannels [10]; however, the AWJM process suffers from poor surface texture such as striation marks and the grit embedment issues [9], and tapered geometry of machined features [11,12].

The WEDM process employs the discrete electrical sparks to erode the workpiece material via melting and evaporation phenomena as detailed in [13]. The wire is continuously fed to the machining zone, and a computer controls its motion path. WEDM can be used to machine deep holes and slots where other processes cannot be successful [14,15]. For example, Bae et al. [16] used WEDM to produce microgrooves with a width of 500 μm and various depth sizes on stainless steel. Jafari et al. [17] fabricated rectangular microchannel evaporators by using WEDM with dimensions of 700 μm height, 250 μm width, and 19 mm length. Zhou et al. [7] used the WEDM process to produce micro-grooved structures (V-shape) with a width of less than 100 µm and a depth of less than 50 µm on the near-super hydrophobic Ti_3_SiC_2_. Ahmed et al. [18] used the WEDM process to fabricate successive deep microchannels with thin interchannel fins with a designed cross-sectional area of 1000 × 1000 µm in copper.

In the literature, only a few studies have been reported regarding investigating the effect of the WEDM process parameters on Monel 400. Selvakumar et al. [19] studied the effects of the pulse-on time, pulse frequency, peak current and job thickness on the material removal rate (MRR) and the surface roughness when machining Monel 400 alloy with WEDM by using response surface methodology (RSM). They found that all factors have effects on both MRR and surface roughness. They used the Pareto optimality approach for multi-performance optimization. Kumar et al. [20] used RSM to optimize the WEDM process parameters including discharge current, pulse-on time, pulse-off time and servo voltage, and investigated their effects on machining rate and surface roughness when machining Monel 400. They used the desirability function for multi-performance optimization. It was concluded that all four considered parameters significantly affect both MRR and surface roughness. Kumar and Babu [21] developed a WEDM process which utilized a minimum quantity of the air and water as mist instead of a conventional high flow rate of water-based dielectric fluid. They employed their mist based dielectric setup to machine slots in Monel 400 alloy. The effects of pulse-on time, pulse-off time, wire feed, air inlet pressure, and water flow rate on material removal rate, surface roughness, and environment were investigated through RSM. They found that pulse-on time, pulse-off time, and voltage are the most significant factors that affect the MRR and surface finish. Jangra et al. [22] conducted a comparative study of rough and trim cutting during the WEDM of WC–Co composite, HCHCr steel alloy, Nimonic 90 and Monel 400 using a multi-cut strategy. It was stated that surface characteristics could be improved when a single trim cutting operation with appropriate wire offset is used irrespective of the previous rough cutting operation.

It should be noted that all the above-discussed studies are focusing only on the through-cutting of Monel 400 by using the WEDM process. Until present, no study has been reported to machine microchannels in Monel 400 alloy by WEDM, whereas it has been discussed that Monel 400 has high potential in micro-heat exchangers. Patil and Mudigonda [2,5] machined microchannels in Monel 400 by using the photochemical machining process. However, it has been mentioned previously that the PCM process is not environment-friendly and is only favorable for high production rates. The current work will focus on the capacity of the WEDM to produce the microchannels in the Ni-based Monel 400 alloy by using the one-factor-at-a-time approach. The effects of WEDM process parameters, including pulse-on time, pulse-off time, average gap voltage, wire feed, and dielectric flow rate, are taken into account. The microchannels are evaluated by dimensional accuracy, machining speed, surface roughness, surface morphology, microhardness and microstructure.

## 2. Materials and Methods

**Figure 1:**
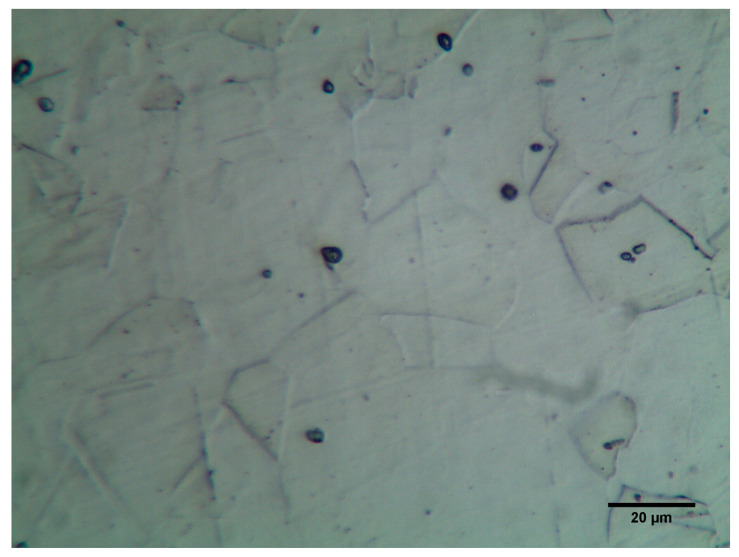
Monel 400 microstructure.

The experiments were conducted on the Electronica ECOCUT CNC wire electrical discharge machine manufactured by Electronica Machine Tool Ltd. (Pune, India). In this study, a rectangular plate of nickel-based alloy, namely Monel 400 (Osaka Stainless Co. Ltd., Osaka, Japan), was used with dimensions of 50 × 20 × 5 mm. Monel 400 alloy is a nickel-copper alloy. It is a single-phase alloy. The chemical composition and mechanical properties of Monel 400 are shown in Table 1 and Table 2, respectively. The microstructure of Monel 400 is shown in Figure 1.

**Figure 2:**
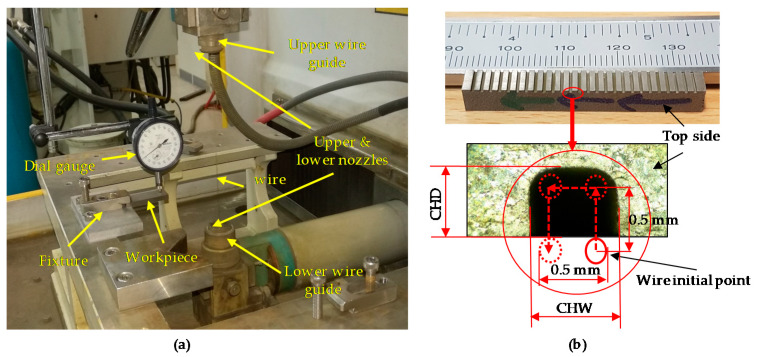
Machine setup (**a**) workpiece fixing and leveling (**b**) wire electrode path profile, not to scale.

The workpiece plate was fixed using an aluminum fixture. Since we are producing microchannels, to ensure the workpiece perpendicularity to the machine axes, a dial gauge was used to level the workpiece in the range of ±2 µm (see Figure 2a). A 1 mm distance was left between the adjacent microchannels to ensure that the heat-affected zone of the preceding microchannel should not affect the neighboring channels (see Figure 2b). Hard brass wire with a diameter of 0.25 mm was used in all experiments as wire material. Deionized water is continuously flushed through the nozzles on both sides of the workpiece (see Figure 2a). The dimensions of the microchannels were set as 0.5 × 0.5 × 5 mm, which refer to the commonly used dimensions of channels in micro-heat exchangers [23]. Figure 2b shows the rectangular cross-section of the microchannels machined on Monel 400 alloy using WEDM and the schematic diagram of the wire path during machining. All the experiments were performed in trim cutting mode with a constant feed rate.

**Figure 3:**
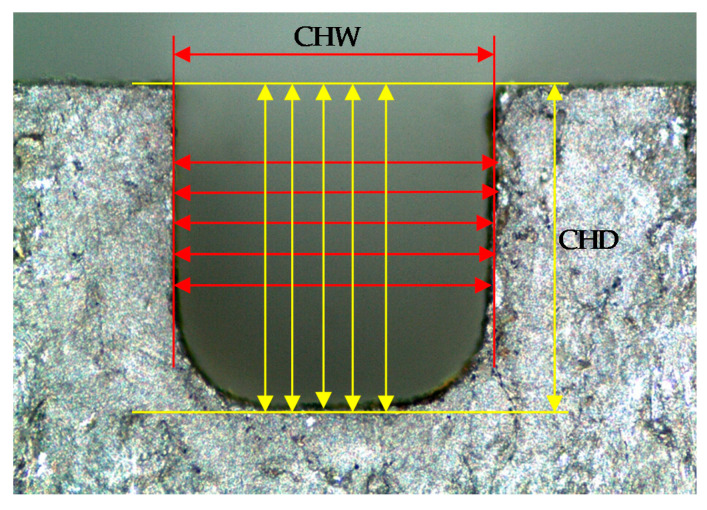
Microchannel dimensional measure (microchannel width (CHW) and depth (CHD)).

**Figure 4:**
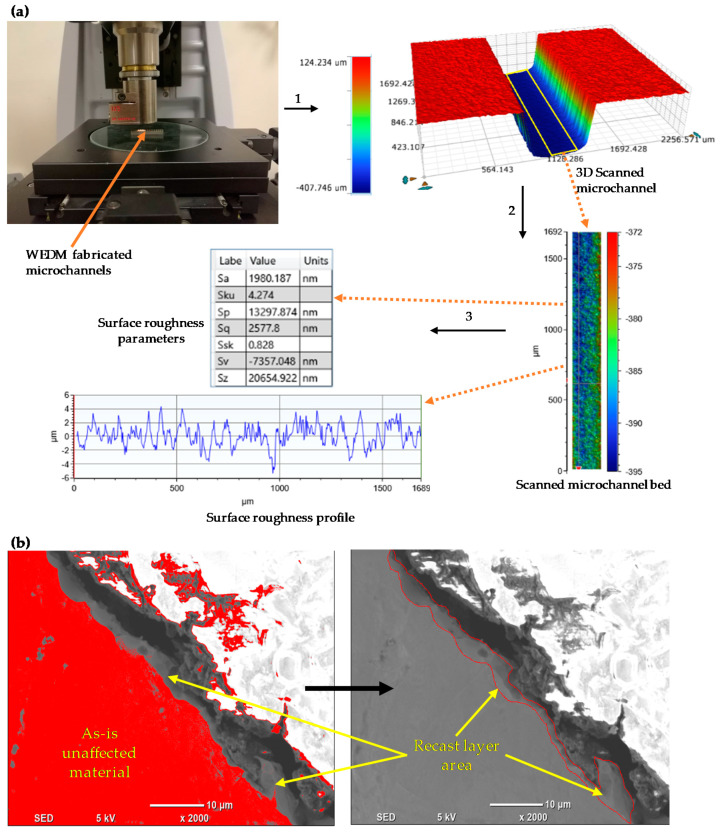
(**a**) procedures for extracting *Sa*, *Sq* and *Sz* and 2D profiles from wire electrical discharge machining (WEDM) machined microchannels, (**b**) measuring the recast layer area from a scanning electron microscope (SEM) image by using ImageJ software.

The average machining speed MS (mm/min) was calculated by dividing the machining path length to the machining time obtained from the stopwatch during machining. Microchannel width (CHW) and depth (CHD) were measured on one side, as in [18,24], the top side, by using a microscope (Mikroskop Technik Rathenow, Rathenow, Germany, MZM 1). Five readings along the microchannel cross-section were recorded, and their average was used to calculate the CHW and CHD as shown in Figure 3. 3D surface scanning of the microchannel beds was performed using a 3D Optical Microscope (ContourGT Profiler) from Bruker, Beerlika, MA, USA. An area of 1.69 × 0.20 mm^2^ (length × width) along the microchannel bed was extracted out of each 3D scanned surface to obtain the surface roughness characteristics namely *Sa*, *Sz* and *Sq*, and the corresponding 2D roughness profiles. A brief illustration of the scanning process is presented in Figure 4a.

Surface morphology was studied by using a tabletop scanning electron microscope (SEM) from JEOL, Tokyo, Japan (Model JCM 6000plus). For microhardness and microstructure analysis, the cross-section of the WEDM machined surface was mounted and then ground with SiC abrasive papers, in which grits were ranged from P160 to P2500. After grinding, the samples were polished with Al_2_O_3_ suspension. Nitric acid (10 mL)–acetic acid (glacial) (10 mL) etchant was used for 7 s. The microhardness measurement was conducted on a Vickers microhardness tester (Struer Durascan, Ballerup, Denmark) at a load of 0.10 kgf with a dwell time of 10 s. The microhardness below the machined surface was measured in a step of 10 µm until 90 µm depth, and every measurement was repeated three times. The first measurement is 20 µm far from the machined surface. For measuring the recast layer thickness, SEM images of the cross-sectioned samples were taken. The average recast layer thickness was calculated as the recast layer area measured from the SEM micrograph divided by the micrograph length, as illustrated in [25]. The area of the recast layer was determined through manual inspection, and later using the ImageJ software (National Institutes of Health, Bethesda, ML, USA). Figure 4b shows an SEM micrograph analysis in ImageJ where the recast layer was first identified by using a threshold and then highlighted in the red highlighted area. It should be noticed that the same threshold level was used for analyzing all the images. The threshold level was initially determined by analyzing an SEM micrograph, in which the recast layer was quite prominent and can easily be identified. The image threshold was selected such that it separated the recast layer area from the unaffected material, as highlighted in red color in Figure 4b.

The effect of the five WEDM process parameters namely, pulse-on time (TON), pulse-off time (TOFF), average gap voltage (VGAP), wire feed (WF) and dielectric flow rate (FR) were studied using one-factor-at-a time approach. Only one input parameter was varied while keeping all other input parameters constant at their intermediate values, except for wire feed, which was held at its lower value to minimize the machining cost while also avoiding the chance of wire breakage. For example, when one input factor is varied within its range, the other factors are kept constant such that TON = 5 MU, TOFF = 5 MU, VGAP = 55V, WF = 5 m/min and DF = 3.5 L/min. Regarding the dielectric flow rate, the same flow rate was used for the upper and the lower nozzles. It is based on the previous studies that reported reduced wire vibrations were observed when the same flow rate is used for both the nozzles [26]. The level of these variables is shown in Table 3. The ranges of input parameters were selected based on the literature and machine capability. Sixteen experiments were performed, and each experiment was repeated three times to consider the experimental error. The mean values of the three replications were calculated and reported herein. Therefore, a total of 48 experiments were conducted on WEDM in trim cutting mode. Furthermore, the standard errors were calculated and displayed by using error bars in the figures.

## 3. Results and Discussion

### 3.1. Parametric Influence on Machining Speed (MS)

**Figure 5:**
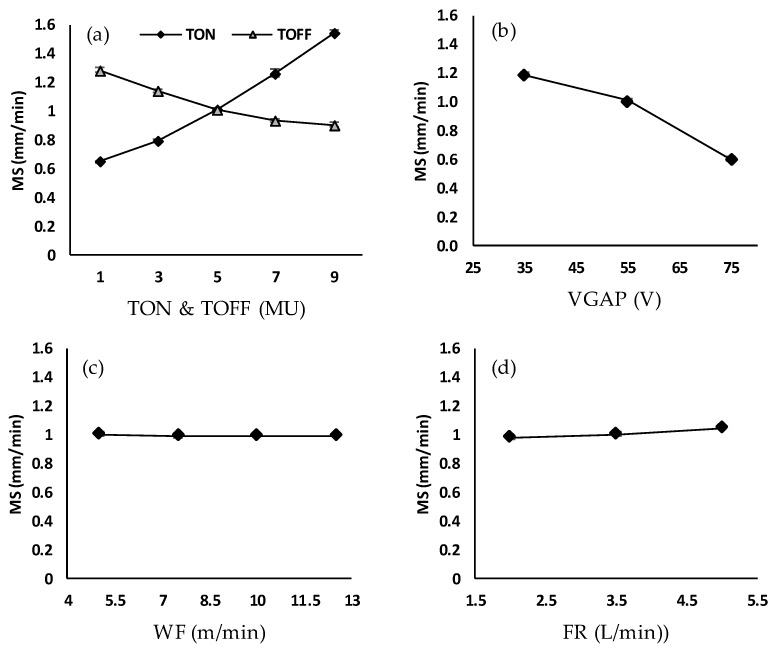
Effect of (**a**) pulse-on time and pulse-off time, (**b**) average voltage gap, (**c**) wire feed and (**d**) flow rate on machining speed (MS).

Machining speed determines the productivity of the WEDM process. Figure 5a–d shows the effects of the WEDM process variables, namely TON, TOFF, VGAP, WF and FR on machining speed. For a fixed TOFF, VGAP, WF, and FR, the MS is directly proportional to the pulse-on time, as shown in Figure 5a. It is because the discharge energy becomes more intense with increased pulse-on times resulting in an increment in the thermal energy per unit time, and thus, augmenting the material removal rate [27]. The machining speed raises from about 0.650 mm/min to about 1.545 mm/min when the pulse-on time increases from 1 to 9 MU. Figure 5a shows that machining speed decreases from about 1.286 mm/min to about 0.903 mm/min when the TOFF increases from 1 to 9 MU. It is evident that an increase in TOFF inevitably raises the idle time, which leads to a drop in the temperature of the machined surface before the next spark begins, thereby resulting in decreased machining speed [28]. Figure 5b illustrates that the increase in VGAP leads to a decrease in MS. The spark gap voltage determines the gap between the wire electrode and the workpiece. Higher VGAP creates a more significant gap between the wire and the workpiece that results in increasing the dielectric-fluid strength. In addition, since the WEDM machine voltage is fixed, the spark current reduces, causing a reduction in the thermal erosion of the workpiece material [29]. As a result, the machining speed is decreased from about 1.184 mm/min to about 0.600 mm/min, with increasing the average gap voltage from 35 to 75 V. Wire feed has no effect on the machining speed when keeping other variables fixed (see Figure 5c). However, it should be noted that the machine frequently stopped at low wire feed rate and those combinations of TON, TOFF and VGAP that leads to high discharge energy. It is because at high discharge energy combined with low wire feed rate the rate of debris deposition on the wire increases which leads to short-circuiting, causing the machine to stop. Therefore, a high wire feed rate is recommended with high discharge energy. Figure 5d shows that the water flow rate has a slight influence on the MS, since Monel 400 alloy has a relatively high thermal conductivity. The minimum and maximum values of the machining speed, 0.601 and 1.546 mm/min, were observed at the highest values of average gap voltage (75 V) and pulse-on time (9 MU) respectively.

### 3.2. Parametric Influence on Microchannels Width (CHW) and Depth (CHD)

**Figure 6:**
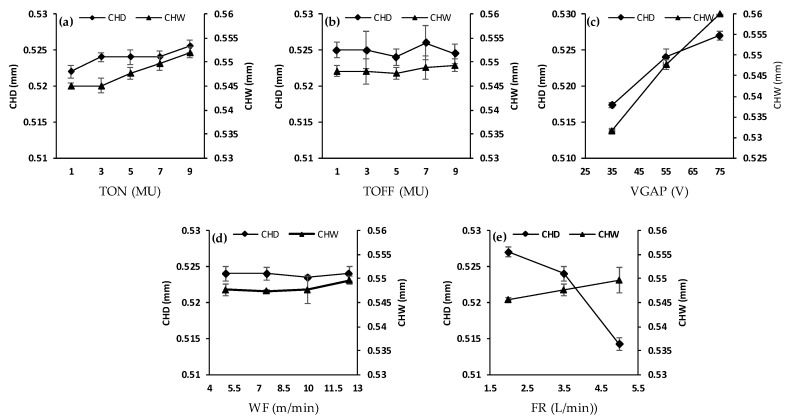
Effect of (**a**) pulse-on time, (**b**) pulse-off time, (**c**) average voltage gap, (**d**) wire feed and (**e**) flow rate on microchannel width CHW and depth CHD.

**Figure 7:**
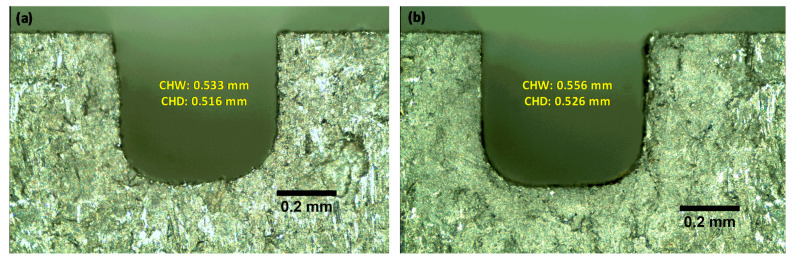
Microchannel width and depth at (**a**) low average voltage (35 V) and (**b**) high average voltage (75 V).

Microchannel width CHW and depth CHD measure the dimensional accuracy of the fabricated microchannels. Figure 6a–e shows the effects of pulse-on time, pulse-off time, average gap voltage, wire feed and water flow rate on the CHW and CHD. It should be noted that the overcut in both CHW and CHD is an inherent characteristic of the WEDM process and could not be avoided; instead, it can only be reduced. Furthermore, it is also noted that the overcut in CHD is close to half of that in the CHW, because the overcut in the channel width occurs on both the side walls, while it only happens once on the channel bed. It seems from Figure 6a that microchannel width increases with the increase in pulse-on time. This is because of increasing the discharge energy with the rise of pulse-on time. Pulse-off time effect on CHW is insignificant compared to other parameters, as shown in Figure 6b. Theoretically, increasing the pulse-off time should lead to a decrease in the CHW and CHD, as the increase in pulse-off time increases the idle time of the machine. However, during the idle time, water flow clears the debris more efficiently from the machined surface, which enhances the erosion from the succeeding sparks on the debris-free cleaned surface. As a result, the effect of the idle time is compensated by the debris cleaning action of the water. It was also observed that the average gap voltage has the most significant effect on the CHW, as shown in Figure 6c. This is due to the fact that the VGAP is a function of the distance between the wire and the surrounding machined surface; the smaller the distance, the smaller the VGAP, and vice versa. Hence, when a higher VGAP is set, it leads to a higher distance between the workpiece and the wire, leading to higher overcut (CHW and CHD). Wire feed (WF) has a slight effect on the CHW and CHD. For example, the microchannel width increases from 0.547 to 0.550 µm when wire feed increases from 5 to 12.5 m/min. The water flow rate shows a slight effect on the microchannel width, as shown in Figure 6e. Increasing the flow rate increases the vibrations in the wire and this leads to an increase in the microchannel width. On the other hand, the CHD significantly decreases with an increase in the dielectric water flow rate (FR). This is due to the reason that as the FR increases, it mounts pressure between the wire and the machined surface, which deflects the wire opposite to the cutting direction [30]. With 90° corner cutting, as in our case, the influence of the dielectric pressure on the corner shape accuracy is large under relatively high flow rate conditions which in consequence leads to decreased channel depth [30] (see Figure 6e). The minimum and maximum values of the CHW, 0.532 and 0.560 µm, were observed at the lowest (35 V) and highest (75 V) values of average gap voltage (VGAP) respectively, while the other factors were fixed. The minimum value of the CHD 0.514 µm was observed at the highest value of flow rate (5 L/min), while the maximum value of the CHD 0.527 µm was noted at the highest value of average gap voltage (75 V) and lowest value of flow rate (2 L/min). Figure 7a–b shows the cross-section of the two microchannels as examples that were machined at low (35 V) and high (75 V) average gap voltage, while the other factors remained constant (TON is 5 MU, TOFF is 5 MU, WF is 5 m/min and DF IS 3.5 L/min). At low average gap voltage (35 V), the width and depth of the microchannel were 0.533 mm (at its lowest value) and 0.516 mm, respectively, as shown in Figure 7a. In contrast, the microchannel width and depth at high average gap voltage (75 V) were increased to 0.556 mm (its highest value) and 0.526 mm, respectively, as shown in Figure 7b.

### 3.3. Parametric Influence on Surface Roughness (Sa, Sq and Sz)

**Figure 8:**
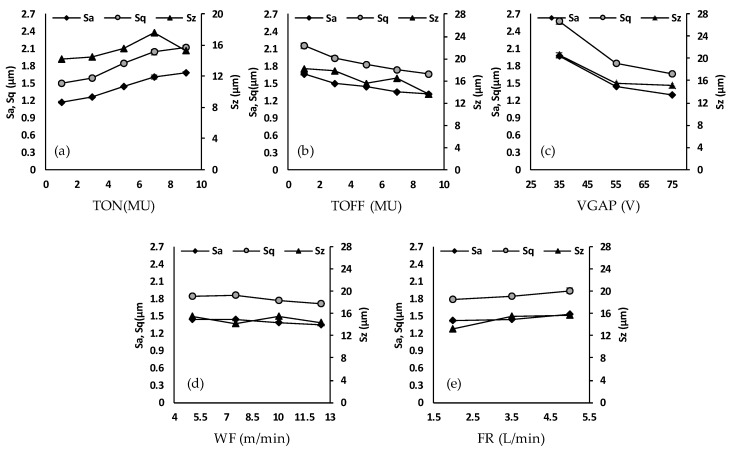
Effect of (**a**) pulse-on time, (**b**) pulse-off time, (**c**) average voltage gap, (**d**) wire feed and (**e**) flow rate on surface roughness (*Sa, Sq* and *Sz*).

**Figure 9:**
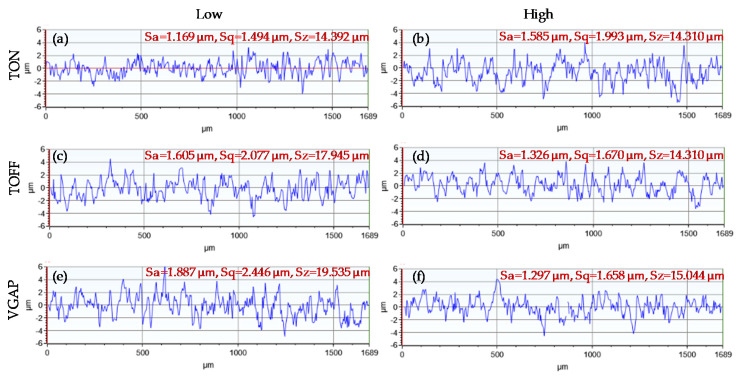
Roughness profiles of (**a**) low TON (1 MU) (**b**) high TON (9 MU) (**c**) low TOF (1 MU) (**d**) high TOF (9 MU) (**e**) low VGAP (35 V) (**f**) high VGAP (75 V).

The surface roughness of the microchannels has a significant effect on the heat transfer characteristics, pressure drop and velocity fluctuations. An increased surface roughness will result in an increased pressure drop and velocity fluctuations [31]. Surface roughness characteristics including arithmetical mean height (*Sa*), root mean square roughness (*Sq*) and maximum height (*Sz*) are studied. Figure 8a–e shows the effects of pulse-on time, pulse-off time, average gap voltage, wire feed and water flow rate on the surface roughness characteristics, namely *Sa*, *Sq* and *Sz*. It can be seen in Figure 8a that the increase in pulse-on time from 1 to 9 MU increases *Sa, Sq* and *Sz*. It is because high discharge energy is generated with an increase in pulse-on time, which leads to the formation of wider and deeper craters on the machined surface. Figure 8b shows that the surface roughness decreases with an increase in pulse-off time. This is due to the formation of a shorter ignition ratio at the longer pulse-off time and thereby forming small craters [32]. In addition, higher TOFF enhances the circulation of the water in the wire-workpiece gap by permitting more time for debris removal before the next spark [32]. Figure 8c reveals that the surface roughness decreases with an increase in average gap voltage. The reason behind it is that lower VGAP results in lowering the wire-workpiece gap/distance. This leads to a reduction in the dielectric strength in the wire-workpiece gap resulting in the intensification of the spark current. Hence, high melting and evaporation of the work material occur. As a result, the surface roughness is increased. In contrast, when a higher VGAP is employed, the flushing condition is improved due to the increased gap between the wire and the material. This consequently leads to splashing the molten metal away from the machined surface, producing fine surfaces. From Figure 8d, it can be inferred that wire feed has a small impact on the *Sa, Sq* and *Sz*. An increase in the wire feed leads to slightly improved surface roughness due to better machining stability. However, a minimum wire feed can be maintained enough to reduce the costs and also to avoid the wire breakage. Dielectric flow rate has a small effect on the studied surface roughness characteristics, an increase in the flow rate leads to a slight increase in surface roughness due to increased wire vibration, as shown in Figure 8e. Overall, from Figure 8, it can be concluded that out of all the studied WEDM parameters, the quality of the machined surface is profoundly affected by the average gap voltage, pulse-on time and pulse-off time. VGAP has the strongest influence on the surface roughness parameters. For example, an increase in the VGAP from 35 to 75 V results in a decrease in the *Sa* from 1.980 to 1.303 µm. After VGAP, TON has the most prominent effect on roughness parameters, for example, an increase in the TON from 1 to 9 MU results in an increase in the *Sa* from 1.174 to 1.688 µm. A smoother surface can be achieved at the low pulse-on time, high pulse-off time and high average gap voltage. Figure 9a–e shows the roughness profiles generated at low and high values of the pulse-on time, pulse-off time and average gap voltage. It is evident from Figure 9 that smooth surfaces can be obtained at the low pulse-on time (Figure 9a), high pulse-off time (Figure 9d), and high average gap voltage (Figure 9f). Rough surfaces, by comparison, are produced at the high pulse-on time (Figure 9b), low pulse-off time (Figure 9c), and low average gap voltage (Figure 9e).

### 3.4. Surface Morphology

**Figure 10:**
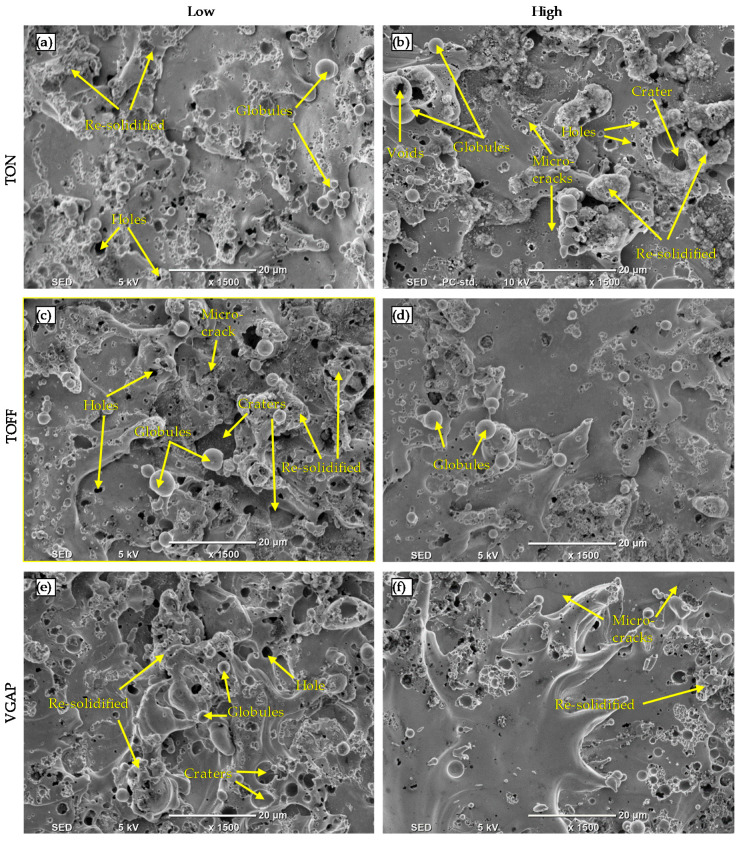
SEM images of WEDM machined surfaces at (**a**) low TON (1 MU) (**b**) high TON (9 MU) (**c**) low TOF (1 MU) (**d**) high TOF (9 MU) (**e**) low VGAP (35 V) (**f**) high VGAP (75 V).

The surface morphology of the microchannels fabricated on Monel 400 by the WEDM process was studied based on the SEM images obtained at a magnification of 1500X. Considering brevity, the SEM images are only presented for the low and high values of those parameters (TON, TOFF, VGAP) which showed a high influence on the surface roughness parameters. From Figure 10a–e, the surface morphology of the microchannels reveals characteristics such as microcraters, microvoids, microglobules, resolidified material, microcracks and microholes, and similar defects were also reported in the previous studies [25,32,33]. The distribution of the microcracks on the WEDM machined surface was small. This is because of the very low percentage of the C element (about 0.12%) in Monel 400 [22].

From Figure 10a–b, it was observed that microglobules, microholes, craters, and resolidified material are more prominent at higher pulse-on time (9 MU) than that of lower pulse-on time (1 MU. Microcracks are also observed only at the high pulse-on time, as shown in Figure 10b. High pulse-on time leads to an increase in the rate of heat energy, and hence, increase the rate of melting and vaporization. This leads to an increase in the size of the craters and redeposited solidified material on the machined surface.

Similarly, microglobules, microholes, craters, microcracks, and resolidified material are more noticeable at the low pulse-off time (1 MU), as shown in Figure 10c. Since the flushing of melted debris at the low pulse-off time is considerably reduced due to reduce cooling time and thus increase the formation of the microvoids, microcracks and microglobules [33]. Increasing pulse-off time increases the flushing of melted material. Moreover, it improves the quality of the WEDM machined surface which can be seen in Figure 10d in which the size of globules, craters, holes and resolidified material are considerably reduced.

Figure 10e shows the WEDM machined surface at a low average gap voltage (35 V), which clearly illustrates the presence of a high volume of the microglobules, microholes, craters, and resolidified material. The molten material is accumulated and resolidified over the machined surface due to an insufficient flushing action owning to the narrow gap size between the wire and the machined surface. Microglobules, microholes, craters, and resolidified material tend to be reduced at high average gap voltage (75 V), as shown in Figure 10f. This is due to the increase of the flushing action which consequently leads to the splashing of the molten material from the surface, which prevents the molten metal to be accumulated and resolidified over the machined surface.

Figure 10a–f shows that the pulse-on time, pulse-off time and average gap voltage have a significant effect on the surface quality of the WEDM machined components. Resolidified material, microholes, and microglobules are significantly reduced at the low pulse-on time, high pulse-off time and high average gap voltage.

### 3.5. Microhardness Analysis

**Figure 11:**
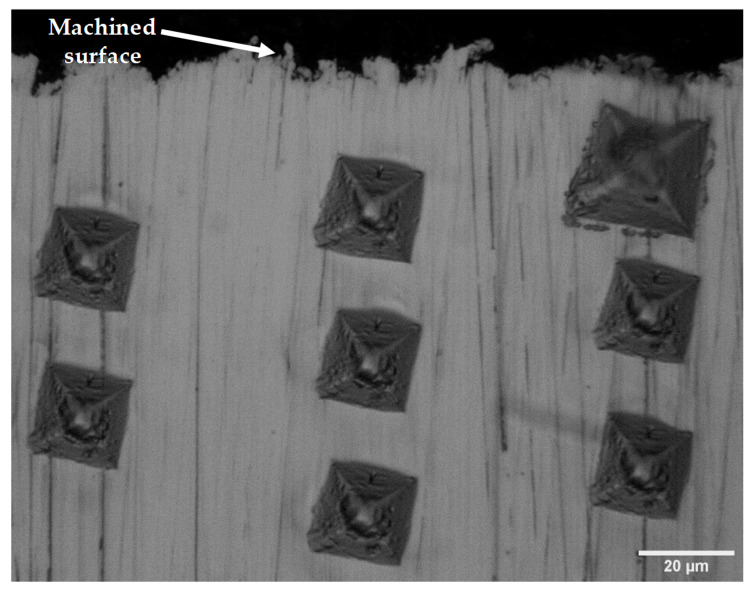
WEDM machined Sub-surface microhardness indents.

**Figure 12:**
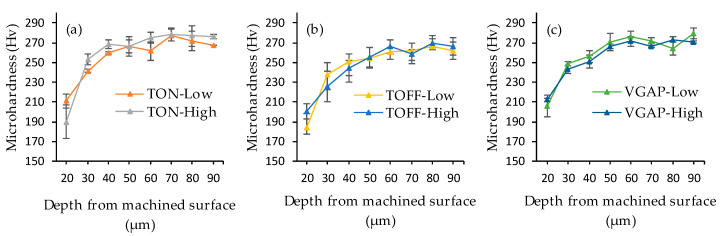
Subsurface microhardness at (**a**) low and high TON (**b**) low and high TOFF (**c**) low and high VGAP.

Figure 11 shows the typical microhardness indents underneath the WEDM machined surface. In general, it can be observed that the size of the indents decreases as the distance beneath the machined surface increases. This can further be seen in Figure 12a–c, which shows the effect of the pulse-on time, pulse-off time and average gap voltage on the microhardness underneath the machined surface. It is clear that the microhardness underneath the WEDM machined surface reduced compared to the bulk material, and this heat affected zone extended generally up to 40 µm. These outcomes are in agreement with the previous studies that suggested a considerable decrease in the sub-surface material hardness subsequent to WEDM [34]. Since Monel 400 has a high percentage of copper (about 32.6%), the thermal conductivity of Monel 400 is relatively high. As a result, a fraction of heat transferred toward the bulk material is more and it will make the subsurface softer [20]. At the high pulse-on time and low pulse-off time, a high reduction in microhardness was observed because the workpiece material is subjected to high discharge energy, as discussed by Sharma [33].

### 3.6. Microstructure Analysis of Recast Layer

**Figure 13:**
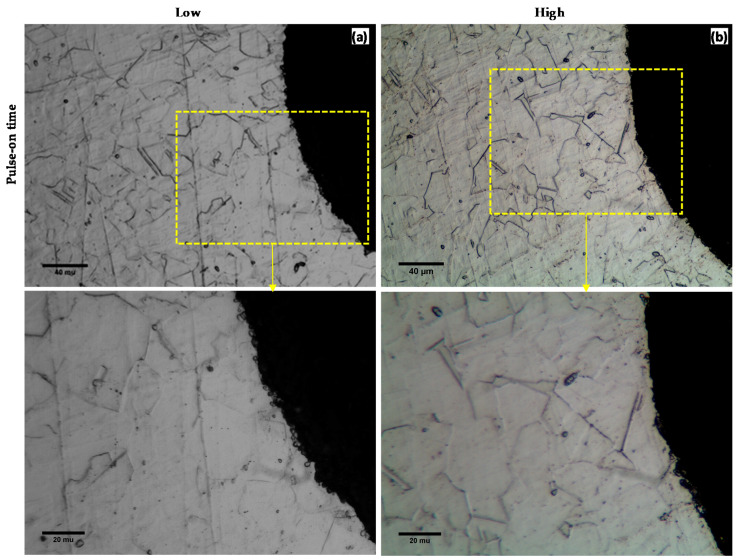
Microstructure of WEDM machined surfaces at (**a**) low TON (1 MU) (**b**) high TON (9 MU).

**Figure 14:**
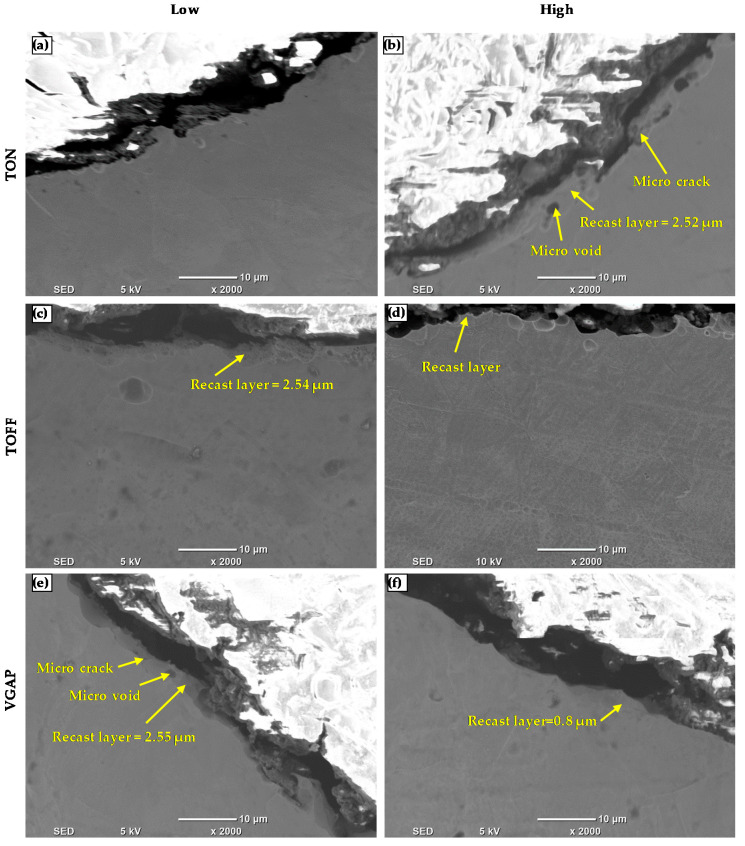
Microstructure of WEDM machined surfaces at (**a**) low TON (1 MU) (**b**) high TON (9 MU) (**c**) low TOFF (1 MU) and (**d**) high TOFF (9 MU) (**e**) low VGAP (35 V) and (**f**) high VGAP (75 V).

The recast layer is the molten material that redeposited on the work surface. Figure 13 presents the microstructure of the microchannel cross-section produced by WEDM at low and high levels of pulse-on and pulse-off times and average gap voltage. Microstructure analysis was conducted by using both the optical microscope (see Figure 13) and the SEM (see Figure 14). Although, the optical microscope provides better microstructural images with visible grain boundaries, as shown in Figure 13. However, the recast layer thickness was so small that the optical microscope could not clearly observe it. This is because the current work involved only trim cutting mode with overall less discharge energy. Therefore, further recast layer analysis was performed on the SEM images as shown in Figure 14. Figure 14a–b shows that the thickness of the recast layer increases with the pulse-on time. Higher pulse-on time provides more energy to the machining area and produces more molten material on the WEDM machined surface and consequently results in a thicker recast layer [34,35]. It is clear that the recast layer thicknesses are not uniform; therefore, the average recast layer thickness is reported [25]. The thickness of the recast layer decreases with the pulse-off time, as shown in Figure 14c–d. Higher pulse-off time provides more time to wash out the molten material from the machining surface, and thus, reduces the recast layer thickness. It was also observed that increasing gap voltage would reduce the recast layer thickness, as shown in Figure 14e–f. This is mainly due to the sufficient flushing action of the molten material owning to the increase in the gap size between the wire and the work surface.

## 4. Conclusions

In this study, the capacity of the WEDM process to produce microchannels on Monel 400 alloy has been studied. The effect of the WEDM process parameters, namely pulse-on time, pulse-off time, average gap voltage, wire feed, and the dielectric flow rate, is investigated. The microchannels were evaluated based on the microchannel depth and width accuracy, machining speed, surface roughness, surface morphology, microhardness and recast layer of Monel 400. From the experimentation and results, the following conclusions are drawn:

1. The microchannel width is significantly affected by pulse-on time and average gap voltage. An increase in pulse-on time and average gap voltage leads to an increase in the microchannel width. Dielectric flow rate has a small effect on microchannel width, in which an increase in flow rate leads to a slight increase in microchannel width.
2. The microchannel depth is mainly affected by pulse-on time, average gap voltage and dielectric flow rate. An increase in pulse-on time and average gap voltage leads to an increase in the microchannel depth while increasing the flow rate decrease the microchannel depth.
3. Machining speed is increased significantly with increasing pulse-on time and decreased with increasing in pulse-off time and average gap voltage.
4. Surface roughness characteristics (*Sa, Sq* and *Sz*) are mainly affected by pulse-on time, pulse-off time, average gap voltage and dielectric flow rate. Surface roughness parameters increase with an increase in pulse-on time and dielectric flow rate and decrease with an increase in average gap voltage and pulse-off time.
5. The surface morphology is profoundly affected by the pulse-on time, pulse-off time and average gap voltage. A smoother surface with reduced occurrences of craters, holes, globules and redeposited materials could be achieved by employing low pulse-on time, high pulse-off time and high average gap voltage.
6. Reduction in the microhardness beneath the machined surface was found to be the inherent characteristics of the WEDM process due to the presence of the recast layer and annealed heat-affected zone. The extent of the heat-affected zone was found to be in the range of 40 to 50 µm below the machined surface.
7. Microstructure analysis of the recast layer revealed an increase in the thickness of the recast layer with an increase in pulse-on time and a decrease in pulse-off time and average gap voltage. The maximum recast layer thickness was limited to 2.55 µm.

This study showed that TON, TOFF, and VGAP are the most effective parameters in controlling the machining speed and microchannels quality (surface integrity and dimensional accuracy). However, it should be noted that there needs to be a compromise between the machining speed and the quality of the microchannels, as both are oppositely affected by the machine parameters. The results showed that by employing low to middle levels of TON, moderate levels of TOFF, and moderate VGAP, microchannels could be produced in Monel 400 with acceptable machining speed, dimensional errors, smoothed surface morphology, and low recast layer thickness.

## Figures and Tables

**Table 1:** Chemical composition of Monel 400.

Composition	C	Si	Mn	S	Cu	Fe	Ni	Co
Weight %	0.12	0.1	1.0	0.00	32.6	2.0	64.1	0.04

**Table 2:** Monel 400 mechanical properties.

Property	Value	Unit	Property	Value	Unit
Yield strength	313	MPA	Thermal conductivity	21.8	W/(m·K)
Tensile strength	565	MPA	Electric Resistivity	54.7 × 10^−8^	Ohm·m
Density	8.8	g/cm^3^	Coefficient of thermal expansion	13.9 × 10^−6^	°C^−1^
Melting point	1350	°C	Specific heat capacity	427	J/(kg·K)

**Table 3:** Wire electrical discharge machining (WEDM) process factors and their respective levels.

Factors	Levels				
Pulse-on time TON, Machine Unit MU (equivalent microseconds)	1 (10)	3 (16.667)	5 (23.333)	7 (30)	9 (36.667)
Pulse-off time TOFF, Machine Unit MU (equivalent microseconds)	1 (25)	3 (41.667)	5 (58.333)	7 (75)	9 (91.667)
Average gap voltage VGAP (V)	35	55	75		
Wire feed WF (m/min)	5	7.5	10	12.5	
Dielectric flow rate DF (L/min)	2	3.5	5		
